# Meteorologically Driven Simulations of Dengue Epidemics in San Juan, PR

**DOI:** 10.1371/journal.pntd.0004002

**Published:** 2015-08-14

**Authors:** Cory W. Morin, Andrew J. Monaghan, Mary H. Hayden, Roberto Barrera, Kacey Ernst

**Affiliations:** 1 Earth Science Office, NASA Marshall Space Flight Center, Huntsville, Alabama, United States of America; 2 Research Applications Laboratory, National Center for Atmospheric Research, Boulder, Colorado, United States of America; 3 Entomology and Ecology Activity, Dengue Branch, Centers for Disease Control and Prevention, Calle Cañada, San Juan, Puerto Rico; 4 Mel and Enid Zuckerman College of Public Health, University of Arizona, Tucson, Arizona, United States of America; Santa Fe Institute, UNITED STATES

## Abstract

Meteorological factors influence dengue virus ecology by modulating vector mosquito population dynamics, viral replication, and transmission. Dynamic modeling techniques can be used to examine how interactions among meteorological variables, vectors and the dengue virus influence transmission. We developed a dengue fever simulation model by coupling a dynamic simulation model for *Aedes aegypti*, the primary mosquito vector for dengue, with a basic epidemiological Susceptible-Exposed-Infectious-Recovered (SEIR) model. Employing a Monte Carlo approach, we simulated dengue transmission during the period of 2010–2013 in San Juan, PR, where dengue fever is endemic. The results of 9600 simulations using varied model parameters were evaluated by statistical comparison (r^2^) with surveillance data of dengue cases reported to the Centers for Disease Control and Prevention. To identify the most influential parameters associated with dengue virus transmission for each period the top 1% of best-fit model simulations were retained and compared. Using the top simulations, dengue cases were simulated well for 2010 (r^2^ = 0.90, p = 0.03), 2011 (r^2^ = 0.83, p = 0.05), and 2012 (r^2^ = 0.94, p = 0.01); however, simulations were weaker for 2013 (r^2^ = 0.25, p = 0.25) and the entire four-year period (r^2^ = 0.44, p = 0.002). Analysis of parameter values from retained simulations revealed that rain dependent container habitats were more prevalent in best-fitting simulations during the wetter 2010 and 2011 years, while human managed (i.e. manually filled) container habitats were more prevalent in best-fitting simulations during the drier 2012 and 2013 years. The simulations further indicate that rainfall strongly modulates the timing of dengue (e.g., epidemics occurred earlier during rainy years) while temperature modulates the annual number of dengue fever cases. Our results suggest that meteorological factors have a time-variable influence on dengue transmission relative to other important environmental and human factors.

## Author Summary

Numerous studies have investigated meteorological and climatic influences on mosquito transmitted viruses. However, dengue ecology is complex, necessitating an understanding of the interactions among components in the system. We estimate dengue fever cases in San Juan, Puerto Rico using a mathematical model informed by relationships among meteorology, land cover, and interactions among human hosts, mosquitoes, and the dengue viruses identified from the literature. Because some of these relationships are not well known or static, we performed several thousand simulations and compared model output to dengue fever cases reported to the Centers for Diseases Control and Prevention. The model replicated reported dengue cases well, but factors related to dengue transmission patterns varied between years. During wetter years, precipitation-filled containers were the primary immature mosquito habitat in the model. Conversely, during drier years, containers filled with water by humans were the most important habitat. In warmer years there was an increased number of dengue cases that peaked following higher rainfall. These results reveal that current climatic conditions modify the relative influence of human and climatic factors on dengue transmission patterns. This knowledge can be used to develop forecasting tools for dengue outbreaks and enhance mosquito control campaigns based on weather predictions.

## Introduction

In the last decade dengue infections have increased dramatically in the Americas with cases now occurring in the southern U.S., Mexico and Central America, across the Caribbean, and as far south as Argentina in South America [1]. The Pan American outbreak in 2010 resulted in 1.7 million cases of dengue fever (DF) including 21,206 cases in Puerto Rico [1]. There are four serotypes of the dengue virus (DENV), and subsequent infection with a new serotype increases the risk of severe dengue which can manifest as hemorrhagic fever (DHF). While DHF case-fatality is fairly low, ranging between 0.5% and 5.0% [2], the global burden of DENV infection is extremely high with an annual estimate of 390 million infections of which 96 million result in symptomatic disease [3]. Given the high burden of disease and that transmission is directly and indirectly regulated by meteorological factors, understanding how dengue dynamics may shift under different meteorological conditions is a key public health question [4–6].

Meteorological factors influence many components of DENV ecology, most directly through the *Aedes (Ae*.*)* mosquito vector [7]. Temperature and precipitation are important drivers of mosquito population dynamics [8]. Temperature influences development rates, mortality, and reproductive behavior [9–12]. Precipitation often provides water in containers that serve as larval and pupal habitat. Container water volume influences development rates and low water volume can increase mortality through enhanced competition between larvae and pupae at higher population densities [13,14]. Ambient air temperature also influences viral replication within the adult mosquito and is a key regulator of the length of the extrinsic incubation period (EIP), the time between when a mosquito is infected and becomes infectious [15–17]. A shorter EIP reduces the length of the cycle of transmission and increases the probability of its completion during the lifespan of *Ae*. *aegypti*.

Despite the established connections between meteorological variables and many components of DENV ecology, the relative influence of weather versus human factors on dengue epidemics is still unclear. For example, although much of the southern United States is inhabited by the vector *Ae*. *aegypti* and lies in close proximity to areas where dengue is endemic, locally acquired infections are rare. Reiter at al. [18] contend that interactions between the vector and human are limited in Laredo, Texas as compared to across the border in Nuevo Laredo, Tamaulipas, Mexico due to better infrastructure which prevents vector-human contact and, thus, limits transmission. Other studies have also found human-related factors to be of greater importance than climatic suitability. Keating [19] and Brunkard at al. [20] both found meteorological variables to influence DF cases but suspect that herd immunity, circulating serotype, and strain play a larger role in transmission. Additionally, human responses to climate may be as important as climate itself. In Australia, Beebe at al. [21] and Kearney at al. [22] found that DENV vector habitat may increase due to human water storage designed to combat drought. The complexity and uncertainty of the many factors involved in the disease system make predictions and effective interventions to reduce transmission difficult.

Process-based models can be useful in determining the relative influence of human versus meteorological factors on DENV transmission. Vector dynamics are frequently tied to variations in temperature and precipitation [23]. Dynamic models are built using known biophysical relationships between the vector, virus and the environment. Mathematical relationships are derived from studies on rates of vector development, mortality, and generational progression as well as thermal limits for population survival [11,24]. Vaidya et al. [25], for example, built a model of mosquito population dynamics using temperature dependent maturation and mortality rates and precipitation dependent carrying capacity. Such models have successfully used meteorological inputs to simulate vector-borne disease dynamics [26–28] including models for *Aedes* mosquitoes that are the vectors of dengue [29,30]. Other uses include exploration of outbreaks based on weather scenarios, and investigations of land use/cover change, and the evaluation of intervention strategies [31]. Because many parameters involved in DENV transmission are unknown, the ability to perform simulations under a variety of conditions and scenarios is an important and powerful aspect of dynamic models that can help characterize parameter uncertainty and whether parameter values vary in time.

Process-based models that simulate vector dynamics are rare and most do not calculate the EIP nor do they simulate transmission between the vector and human populations, in large part due to the complexity of the single and combined models [7]. But vector density does not always correlate with disease incidence [6,32]. A review of dengue transmission modeling approaches by Andraud et al. [33] promotes the use of models that include a vector component for informing public health policy. One of the first models that integrates the two components (human and vector) was developed by Focks et al. [34]. It integrates a dynamic vector population model (the container inhabiting mosquito simulation model, CIMSiM) with a dengue transmission model (the dengue simulation model, DENSiM). While CIMSiM model outputs have been validated in the field, some parameters remain highly uncertain, emphasizing the importance of developing alternative process-based models of dengue transmission [35]. Skeeter Buster, for example, has expanded on CIMSiM to include population genetics, spatial heterogeneity in habitat availability, and stochastic effects [36]. Andraud et al. [33] and another review by Johansson et al. [37] both identify model parameterization as a key challenge for simulating dengue transmission. However, employing numerous models, or many slightly-differing versions of the same model, allows researchers to explore and quantify uncertainty in the parameter space, and model ensembles have been shown to be more accurate than any single model [38].

In this study we present a new modeling framework that couples a dynamic mosquito life-cycle model calibrated for *Ae*. *aegypti* with a SEIR (susceptible-exposed-infectious-recovered) model for dengue transmission to simulate dengue outbreaks in San Juan, Puerto Rico from 2010–2013 (available for download at https://sites.google.com/site/dymsimmodel/home). This work expands upon the existing Dynamic Mosquito Simulation Model (DyMSiM) [8] by incorporating a virus transmission component and parameterizing the model for the mosquito species *Ae*. *aegypti*. The model incorporates newly collected and consolidated research related to temperature effects on vector survival and development [39] and the dengue virus extrinsic incubation period [17]. Using a Monte Carlo approach, simulations are performed for numerous combinations of parameter values and the results are evaluated using dengue case data from San Juan, PR reported to the Centers for Disease Control and Prevention (CDC) Dengue Branch. The best model simulations over the entire time period and for individual years are analyzed to determine the relative influence of different meteorological and human-mediated factors on DF case numbers and to identify how and why parameter values changed between years.

## Methods

### Location

San Juan is a municipality of Puerto Rico and is located in the northeastern part of the Caribbean island. The population was 395, 326 in 2010 (US Census Bureau, 2010 Census). San Juan has a humid tropical climate with minimal variation in seasonal temperature. Precipitation occurs all year, but it is notably drier during boreal winter and early spring. San Juan is an ideal study location because dengue is endemic in the municipality, and weekly dengue case data are available through CDC ArboNet (USGS, 2014) from 2010–2013. During that time, annual case numbers ranged from 500 (2011) to 919 (2012). These data are crucial for model evaluation.

### Model

**Fig 1:**
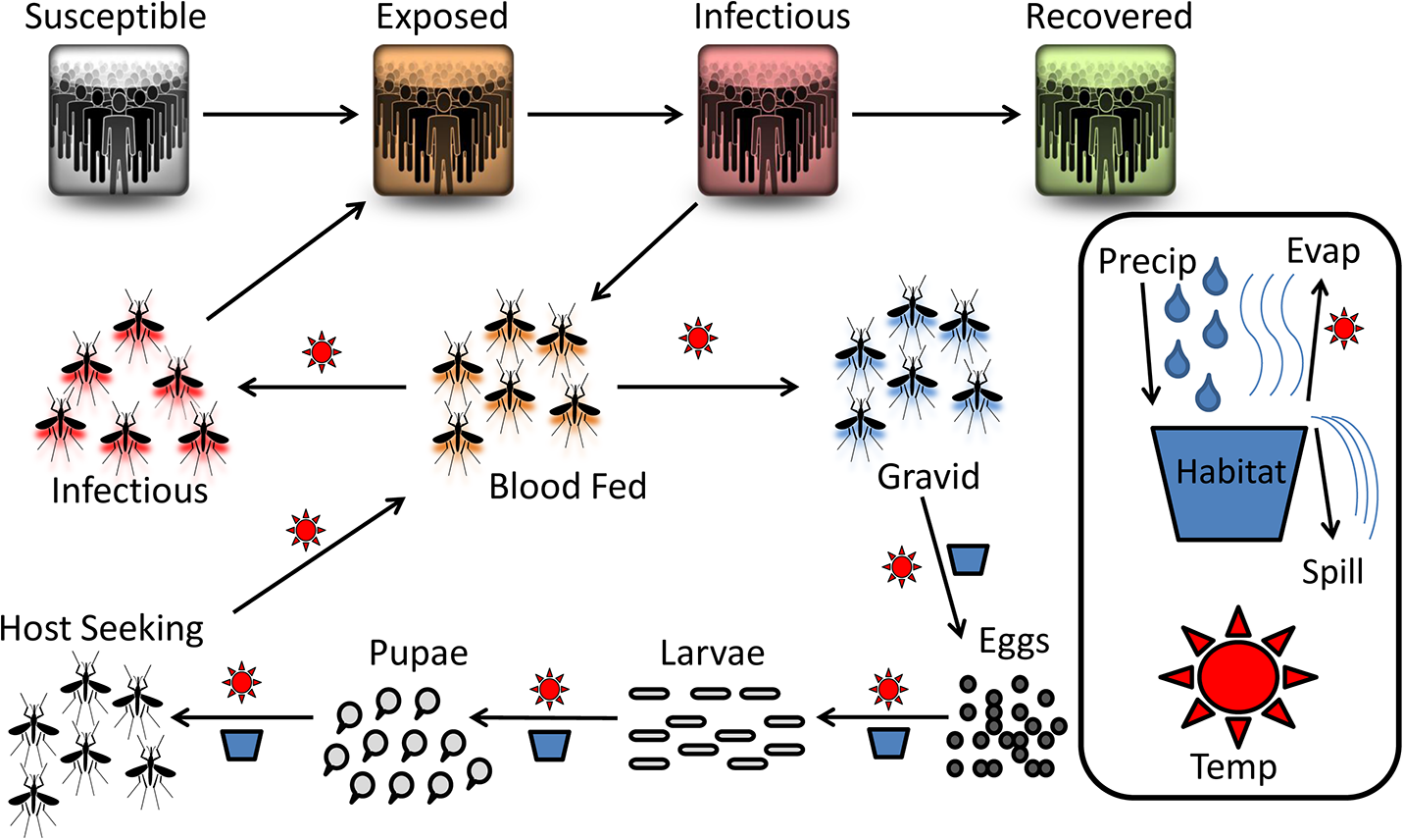
Conceptual diagram of modeled dengue ecology. Suns bordering an arrow indicate that the process is temperature dependent and a habitat/container symbol bordering an arrow indicates the process is habitat/precipitation dependent. Water is added to a habitat through precipitation or manual filling and is lost due to spilling and evaporation which is regulated by temperature. After hatching, the mosquitoes develop through their larval and pupal stages before emerging as adults. The adults blood feed, develop eggs, and then lay them in a water habitat. Upon blood feeding, adults can contract the virus from an infectious human. Those mosquitoes can then expose a susceptible human to the virus during a subsequent blood meal.

The mosquito population was simulated using the general structure of the Dynamic Mosquito Simulation Model, (DyMSiM) [8] but the model was parameterized for *Ae*. *aegypti* mosquitoes with additional components added, including the epidemiological SEIR model (S2 Table). This enabled the simulation of virus transmission between the human and mosquito populations in DyMSiM. The model is deterministic and was implemented using Euler’s method of integration in Stella 10.06 software. A conceptual diagram of model processes is provided in Fig 1. Parameter value constants and equations are provided in S1 Table, governing equations for human and mosquito populations are provided in S2 Table, and further details are discussed below.

#### Immature stages

In the model, cohorts of *Ae*. *aegypti* begin as eggs laid by gravid females (100 eggs per female is used as a reasonable average from the literature [10]). Eggs hatch, develop through their larval and pupal stages and then emerge as adult mosquitoes. Eggs hatch only under suitable air temperatures and when water is available; if water is not available they will go dormant until newly added water initializes hatching. All immature stages are exposed to density independent mortality due to desiccation from inadequate water availability and water temperature sensitivity; the larvae are also exposed to density dependent mortality caused by crowding and lack of food resources. Because water body temperatures remain more constant than air temperatures throughout the day due to water having a higher specific heat than air, development and density independent morality rates during aquatic stages (larva, pupa) are calculated from mean daily air temperature while eggs are calculated from modeled hourly air temperature. The total carrying capacity (i.e., the total amount of immature *Ae*. *aegypti* the system can accommodate) of the system is based on the amount of habitable water in the system (see below) and the effects of crowding on mortality as reported in the literature [13,40]. Density dependent mortality is considered only when the larval population is above the carrying capacity and the survival under those conditions is calculated as the total carrying capacity divided by the larvae population.

#### Adult stage survival and reproduction

Only the female adult mosquitoes (assumed to be 50% of the emerging pupae, although it can vary in nature [12]) are tracked because they take blood meals, lay eggs, and are capable of transmitting the DENV. Females proceed through a cycle of host seeking/feeding, ovarian development, and egg laying (the gonotrophic cycle) until death. It is assumed that all females have eggs that become fertilized within 3 days of emergence unless limited by high or low air temperatures established by Eisen et al. [39], which cause declines in activity [10]. It is assumed that males are always available to mate. Once fertilized, females require a blood meal to gain nutrients for ovarian development. Temperature driven equations governing feeding rates are based upon the literature and observations [10]. The Sharpe and DeMichele [41] enzyme kinetic model, based on ambient temperature and organism specific developmental enzyme coefficients [29], is used to calculate ovarian development rates. Once ovarian development is complete the female will deposit her eggs and then restart the gonotrophic cycle if air temperatures are suitable and a water source is available. Adult survival rates, from mark-release-recapture studies [42–46], are based on daily average air temperature and remain constant except at extreme temperatures (>40°C or <1°C). It is assumed that human hosts are readily available for feeding, female *Ae*. *aegypti* do not feed while gravid, and females retain their eggs when environmental conditions (air temperature or water availability) are unsuitable for laying [10].

#### Adult stage infection

Feeding behavior is partially regulated by air temperature with declines in activity occurring at both high and low thermal extremes. The probability of DENV infection while feeding is based upon the proportion of infectious humans (calculated in the SEIR model) and air temperature [15]. Progress through the EIP for infected females is tracked separately from the gonotrophic cycle. EIP progress (the proportion of the EIP that is complete) is calculated cumulatively each day by summing the inverse of the EIP length at hourly modeled air temperatures (S1 Table). Data on EIP length was taken from Tjaden et al. [17] who integrated data from several studies. Mosquitoes that have completed the EIP, termed “infectious mosquitoes”, can infect human hosts when they blood feed. The probability of host infection when bitten by an infectious mosquito increases with temperature as demonstrated with data from Tjaden et al. [17] and may be related to higher densities of the virus in the salivary glands at warmer temperatures due to accelerated replication. Watts et al. [15] did observe viral titers to be lower in mosquitoes incubated at lower temperatures though the difference decreased over time. The proportion of infectious mosquitoes in the population determines the number of feeding mosquitoes that can potentially infect susceptible human hosts. This is used to determine human exposure in the SEIR model. It is assumed that dengue infected mosquitoes are well mixed in the population and behave similarly to the non-infected mosquitoes. Because studies have shown that *Ae*. *aegypti* feeds almost exclusively on human hosts, we assumed all blood meals are from humans [47,48]; however, in more rural areas of Puerto Rico domestic animals may also be a significant source of blood meals [49].

#### SEIR model

SEIR models are widely used in epidemiology [50]. Our SEIR model tracks DENV transmission to human hosts under force of infection from one combined serotype. Unfortunately there were not sufficient data on circulating serotypes and their infection/immunity rates within the population to track multiple serotypes of the virus. Therefore, we assumed that reinfection with dengue did not occur. Considering the short time period of this study and the low ratio of cases to total population, reinfection is probably rare; however, a longer period of study would require inclusion of infection rates from multiple virus serotypes, rates of re-infection, serotype specific differences in the proportion of asymptomatic infections, and estimates of birth/death and immigration/emigration rates. Cross immunity among serotypes has also been demonstrated over a 1–3 year time interval consistent with the length of this study [51]. Susceptible humans become exposed at a rate determined by the number of infectious blood feeding mosquitoes and the host infection probability [17] (governed by air temperature).When calculating the number of infectious feeding mosquitoes in the vector model, only mosquitoes that have completed one gonotrophic cycle are counted because those mosquitoes taking a first blood meal are incapable of infection with the virus. The length of the exposed stage (latent period when a human is infected but cannot re-infect a feeding mosquito) and infectious stage (period when infected humans can re-infect a feeding mosquito that is taking a blood meal) are highly variable and thus average values were chosen based on approximate numbers reported in the literature [52–54]. The percent of humans in the infectious stage is used to determine the probability that a mosquito takes an infectious blood meal in the vector component of the model. During periods of low host infections, the model assumes a background host infection rate (S1 Table) to sustain DENV transmission to the vector. Once the infectious stage is complete and the host moves to the recovered stage they can no longer become infected.

#### Environmental inputs

Inputs for the environmental component of the model include daily minimum, maximum, and mean air temperature, daily total precipitation, latitude, and container habitat area, height, and composition. As stated earlier, the mean air temperature is used for larval and pupal development rates as well as mortality rates in all vector stages. The gonotrophic cycle, egg development, and dengue viral replication rates, however, are based on hourly temperatures modeled using a sinusoidal wave with an amplitude equal to the diurnal air temperature range calculated from the input daily minimum and maximum air temperature.

Container habitat area is the area in which water can collect via precipitation to form habitat for the immature mosquitoes (larvae/pupae). There are two types of container habitats in the model: precipitation dependent containers, which fill from precipitation and empty via evaporation and spilling, and human managed containers, which are manually filled and are thus subject to much less evaporation, making them semi-permanent. Container habitat composition is simply the proportion of each habitat type comprising the total container habitat area. Water levels within each habitat are calculated by adding precipitation and subtracting evaporation and spilling, which occurs if the amount of water exceeds the volume of the habitat (area x height). The evaporation rate is calculated using Hamon’s equation [55] based on air temperature, saturation vapor pressure, and daylight hours. While saturation vapor pressure is calculated from the daily mean air temperature, daylight hours are calculated using Schoolfield's model which requires latitude as an input [56]. The amount of habitable water in the system is calculated as only the top 1 cm of water in the combined habitat types due to the need for the larvae and pupae to access the surface for air. Total carrying capacity is calculated from the carrying capacity (larvae/cm^3^) multiplied by the amount of habitable water.

### San Juan, PR Simulations

Because it is difficult to obtain precise measurements for many of the DyMSiM parameters, especially those related to immature container habitat, we used a Monte Carlo approach to assess outcomes for a range of possible parameter values. We performed 9600 simulations with DyMSiM using discrete parameter values representative of the distribution of values identified in the literature and the preliminary model simulations (Bold in Column 3 of S1 Table) to replicate DENV transmission in San Juan, Puerto Rico from 2010–2013. During each simulation, one value was changed until all parameter value combinations were used. All humans and mosquitoes were assumed to be susceptible at the beginning of the runs, however, runs were started in 2009 to provide spin-up time to build infection and immunity (human only) within the human and mosquito population. Additionally, a background infection rate was used to initiate infections and is based off of the average number of dengue cases that occur during the low transmission season according to the 2010–2013 case data for San Juan. The assumption that the population is completely susceptible is not accurate, however, given the low incidence (3192 total cases over a population of 395,326) and short time interval over which the model is run, the influence on outcomes should be minor. Weekly CDC reported case data were used to evaluate the model.

Container habitat area estimates were determined by first performing preliminary model simulations under parameter values that would produce a maximum and minimum number of dengue cases in San Juan. By comparing these runs with the reported dengue data, an estimated range of habitat area was determined for the study. Because there is great uncertainty in the container habitat area and it strongly influences mosquito population size, the number and range of values chosen greatly exceeds that of the other parameters. Fewer values and smaller ranges were chosen for parameters where more research has been performed resulting in greater confidence in the selected values.

#### Dengue validation dataset

Weekly confirmed DF cases for San Juan were obtained through ArboNET with data provided by the CDC—National Center for Emerging and Zoonotic Infectious Diseases. The surveillance system is passive. All reported cases must first seek care, be diagnosed by clinicians using appropriate criteria and laboratory tests, and those providers and/or laboratories must report the dengue cases in order for cases to be confirmed. Since the model simulates epidemics with daily time steps and CDC dengue case data are reported in epidemiological weeks, modeled dengue cases (humans moving from the exposed stage to the infectious stage when symptoms typically occur) were aggregated to weekly intervals using dates matching the epidemiological weeks used by the CDC.

#### Meteorological inputs

Surface meteorological data used to drive the model were obtained for San Juan, PR at the Luis Munoz Marin International Airport (GHCND: RQW00011641) from the National Climatic Data Center (NCDC) (http://www.ncdc.noaa.gov/oa/land.html). Meteorological variables included daily maximum and minimum temperature and daily total precipitation for the years 2009–2013 (S1 Dataset). The year 2009 was simulated to allow for model spin-up but the output data was not used for model evaluation or other analysis. Additional San Juan specific input included population from the US Census Bureau and latitude (S1 Table).

#### Comparing simulation and case data

To compare the simulation and CDC case data, the output data was standardized using Eq (1) below:

(MDeni1N∑MDen)*(1N∑RDen) (1)

*MDen* is weekly modeled DF cases for week *i* and *RDen* is CDC reported DF cases. The procedure was performed on the whole time period and repeated for the individual years. Lastly, both the CDC reported and modeled dengue case data was smoothed using a three week moving window. This was done because the model assumes an average time lag between infection and symptom onset and treatment (latent period), however, there exists variability in symptom onset and severity and reporting efficiency[52,54].

Case data were used to analyze model output using two methods. For the first method (which is the primary focus of this paper) the r^2^ value between the reported DF case data and individual model simulations (n = 9600) was used to determine the top 1% (n = 96) of simulations to retain for further analysis by ranking them (1 best and 9600 worst) based on their r^2^ values for individual years and overall. A model ensemble mean was calculated by averaging the simulated standardized weekly case counts from all retained simulations (n = 96). The parameter values used in the simulations that best fit the case data (top 1%) were assessed for patterns that might explain differences in driving transmission for each individual year and overall. Additionally, the average ranks for simulations using each parameter value were calculated from the full 9600 simulations to determine the sensitivity of the model to changes in that parameter and to identify the most influential values for each time period (2010, 2011, 2012, 2013, and overall). For the second method, overall accuracy (r^2^) was determined using all simulations (n = 9600) with all possible parameter value combinations to determine accuracy with no a priori knowledge. We tested the fit of the ensemble median of all 9600 simulations to the raw case (r^2^) without standardization. Lastly, weekly climate statistics were calculated for comparison with DF cases and model parameters. A number of statistical measures were used to evaluate the ensemble model performance including root-mean-square-error (RMSE), systematic RMSE, unsystematic RMSE, regression slope, *r* ^*2*^, and Willmott's index of agreement (*d*) [57].

## Results

### Observed Dengue and Climate Patterns

**Fig 2:**
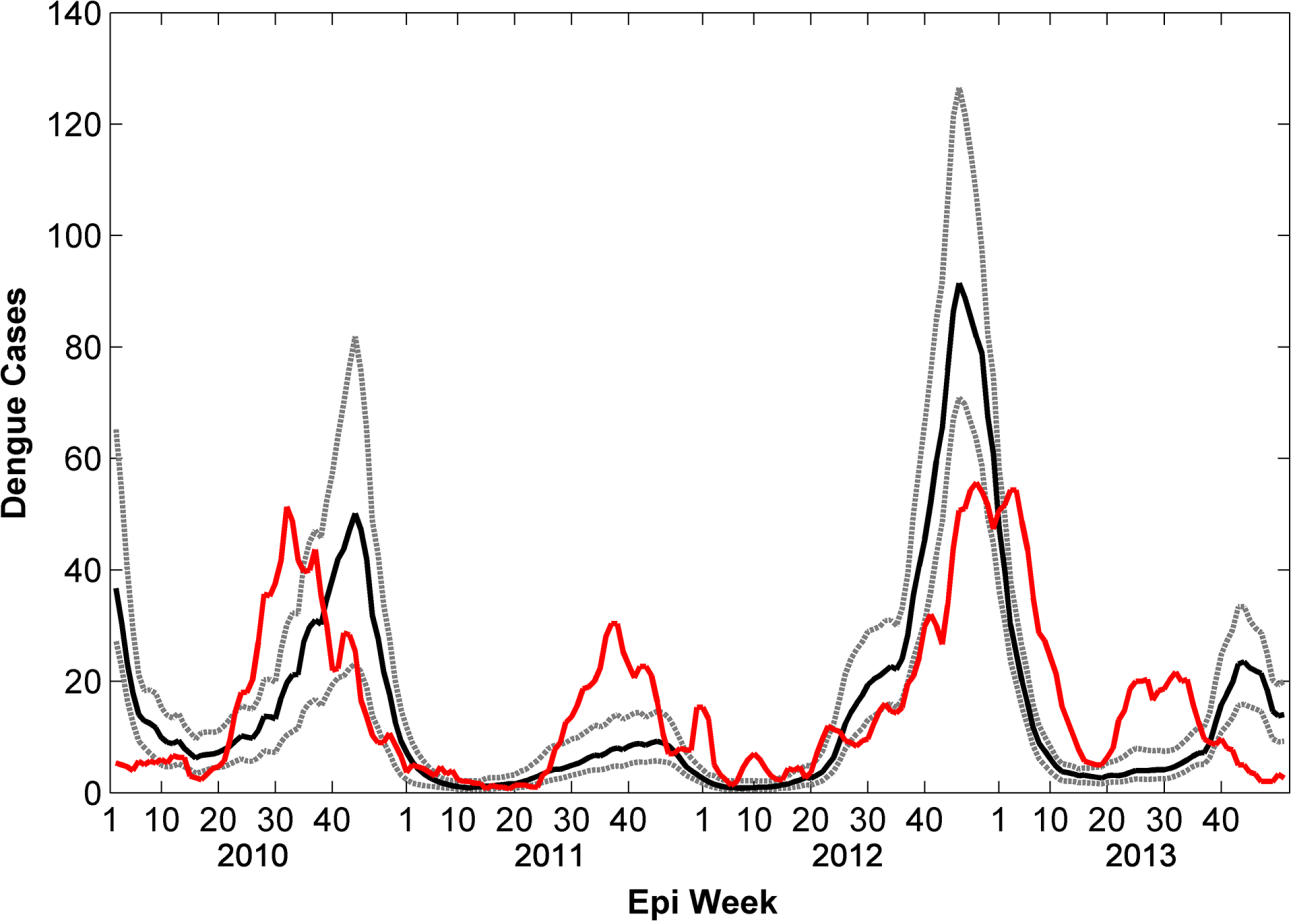
Simulated and reported weekly total dengue fever cases (2010–2013). The model ensemble mean (black line) replicated inter-annual variability in reported DF cases (red line) accurately, however, intra-annual variability is not simulated as well. Dashed gray lines are the ensemble minimum and maximum.

There were 3,192 cases of DF reported during the study period with 901 cases occurring in 2010, 500 cases in 2011, 919 cases in 2012, and 872 cases in 2013. Reported cases began increasing during week 20 in 2010, 2012, and 2013 but occurred later (week 25) in 2011 which was relatively cool compared to the other years and experienced the fewest number of cases (Fig 2). During most years (2010, 2011, and 2013) the peak in reported DF cases occurred between weeks 32 and 37, however, during 2012 the increase in cases was much more gradual and the peak did not occur until week 49 and did not decline to minimum season case numbers until week 15 of 2013. Temperatures were also much warmer during the second half of 2012 compared to other years, while precipitation was notably lower, especially during summer and fall. Although 2013 experienced similar reported case numbers as 2010 and 2012, most cases occurred during the first 15 weeks of the year (Fig 2) with fewer cases occurring during its summer peak. The decline to reported minimum season case numbers also occurred earlier than the other years. Aside from 2012, increases in reported DF followed rains occurring in late spring. The earliest increase and peak in reported DF cases occurred in 2010 which also experienced the warmest spring temperatures and earliest precipitation.

### Comparison of Simulation Results with Surveillance Data

**Fig 3:**
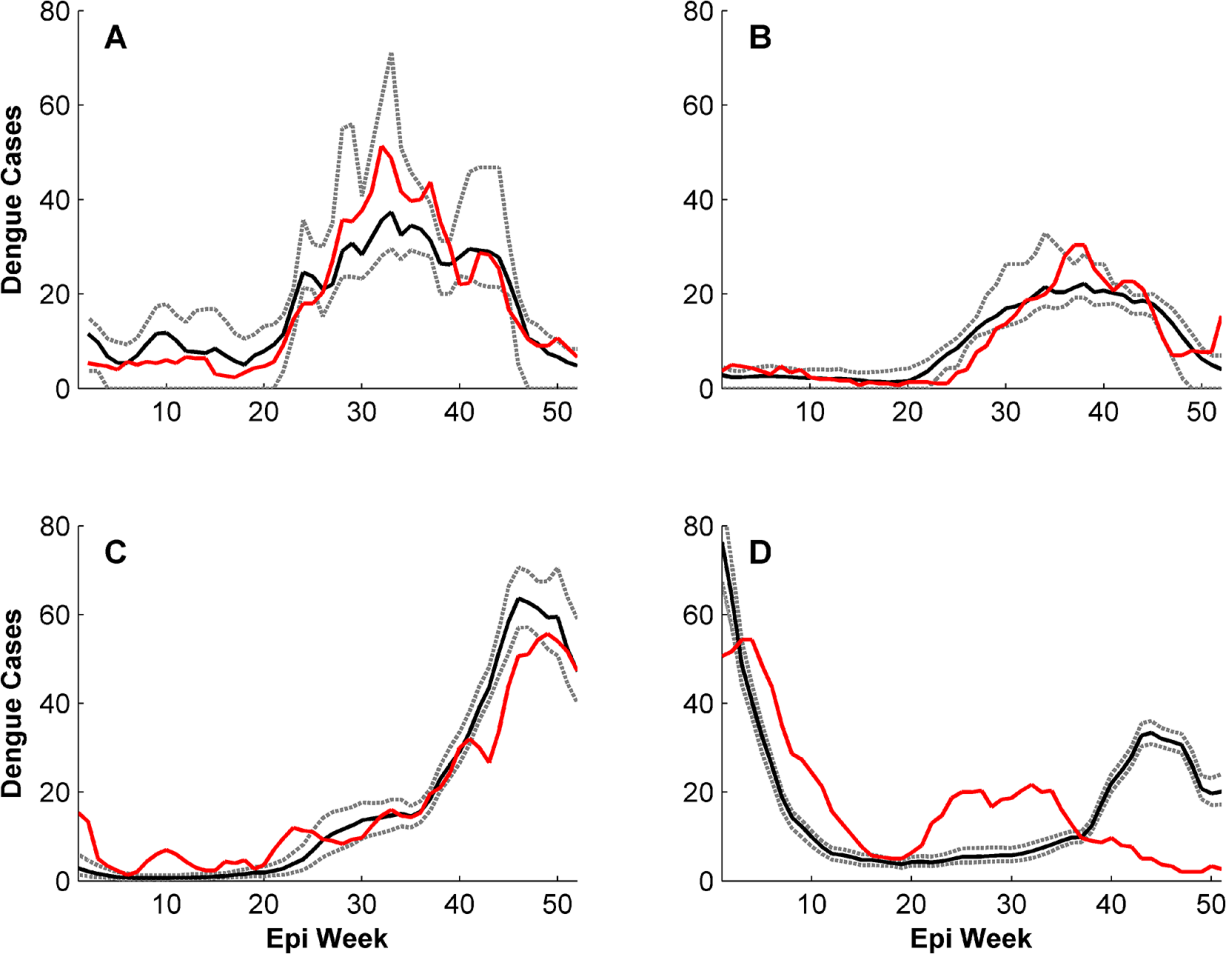
Simulated and reported weekly total dengue fever cases with model parameterized for individual years. The red line is reported DF cases, the black line is the ensemble mean of simulated DF cases, and the dashed gray lines are the ensemble maximum and minimum. Intra-annual variability is captured exceptionally well when the model is parameterized for the individual years 2010(A), 2011(B), and 2012(C). 2013(D) is not simulated as well.

Statistical measures of accuracy between the reported data and ensemble averages using the top 1% (n = 96) best-fit simulations, for both the entire time period (S2 Dataset) and individual years (S3–S6 Datasets), are reported in Table 1. Although inter-annual variability in DF cases was well-simulated in the ensemble average from the 96 model simulations over the entire time period (r^2^ = 0.44, p = 0.002), the annual epidemic curves for individual years often lacked precision (Fig 2). When evaluating ensemble averages from the simulations over individual years (same simulations but the statistics are only performed on reported case data for individual years), however, intra-annual variability was replicated with much higher accuracy for 2010 (r^2^ = 0.90, p = 0.03), 2011 (r^2^ = 0.83, p = 0.05), and 2012 (r^2^ = 0.94, p = 0.01) (Fig 3). Simulations for 2013 were markedly less accurate (r^2^ = 0.25, p = 0.25) compared to the other years (Fig 3). By contrast, the yearly model accuracy assuming no a-priori knowledge to guide parameter selection was lower and more variable: the r^2^ values for 2010, 2011, 2012, and 2013 were 0.77, 0.64, 0.05, and 0.12 respectively.

**Table 1:** Ensemble model validation statistics for the top 1% of simulations for San Juan County, PR parameterized for the entire time period (2010–2013) and individual years.

Year	mean O	mean P	*S*_*o*_	*S*_*P*_	N	a	b	MAE	RMSE	RMSE_S_	RMSE_U_	d	r^2^
**All**	15.46	15.23	14.28	18.77	206	0.87	1.80	10.60	14.21	1.89	14.09	0.79	0.44*
**2010**	17.58	17.43	14.48	10.64	51	0.70	5.20	4.50	5.58	4.41	3.42	0.95	**0.90**
**2011**	9.74	9.61	8.99	7.68	52	0.78	2.05	2.72	3.77	2.01	3.19	0.95	**0.83**
**2012**	17.45	17.67	16.52	20.86	52	1.23	-3.74	4.37	6.20	3.67	4.93	0.97	**0.94**
**2013**	17.16	16.73	14.44	15.67	51	0.54	7.39	12.87	15.07	6.59	13.56	0.73	0.25

Bold indicates significance at p<0.05 and * indicates significance at p<0.01. O = observed, P = predicted, *S* = standard deviation, a = slope, b = intercept, MAE = mean average error, RMSE = route-mean-square error, s = systematic, u = unsystematic, d = Willmott’s index of agreement.

### Parameter Analysis of Model Runs

**Fig 4:**
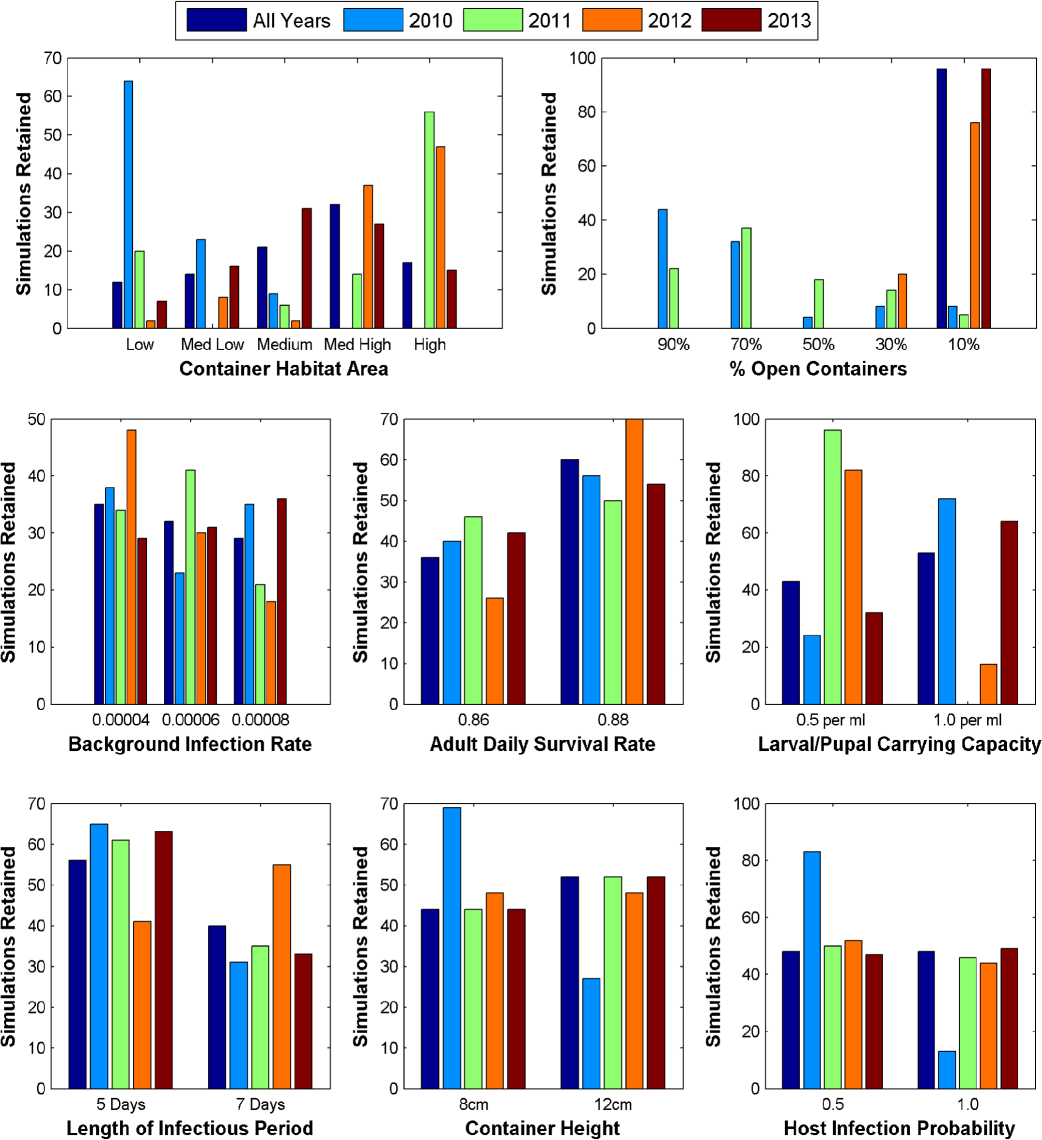
Distribution of parameter values used in the top 1% of simulation for the entire time period (2010–2013) and individual years. The number of times each parameter value is used in the retained simulations is compared between years.

Fig 4 displays the number of times each parameter value was used in the top 1% of simulations. Notably, in 2010 and 2011 the number of simulations retained that used a high proportion of open containers (i.e., more precipitation dependent sources) vs. a low proportion of open containers (i.e., more human-managed sources) is much larger than in 2012 and 2013 (76 and 59 vs. 0 and 0). Additionally, patterns in container habitat area, background infection rate, and carrying capacity vary considerably between years. The retained simulations for most years used the higher adult daily survival rate, and the shorter length of infectious period (except 2012). With the exception of 2011, the range of values tested for container height and host infection probability are used with equal frequency between years indicating that they did not have a strong impact on temporal variation in dengue transmission dynamics.

**Fig 5:**
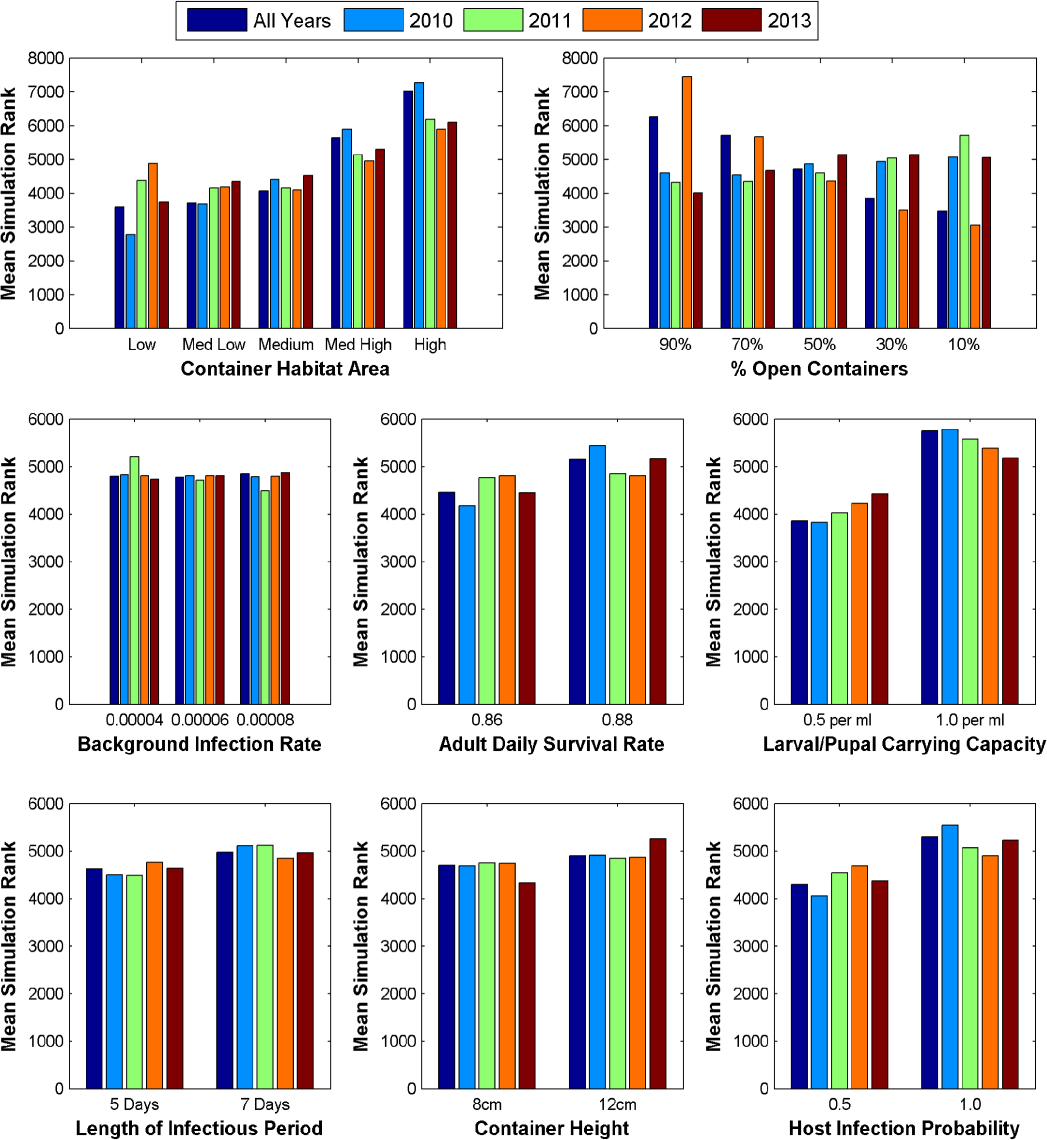
Average ranking of simulations using each parameter value when compared with reported case data for all years and individual years. Rankings were based on r^2^ values with lower value ranks being better and higher value ranks being worse.

Fig 5 displays the average rank of simulations using each parameter value among all 9600 simulations. Simulations that used a high proportion of open containers ranked slightly better for 2011, while the opposite is true for 2012 and all years. This is consistent with the trend in the top 1% of simulations, however, there is no strong difference for 2010 and 2013. Simulations using higher values for container habitat area were markedly worse, though this effect was lesser for 2012. Additionally, the lower carrying capacity value tended to produce better simulations. Average ranks among different values did not vary markedly for the other parameters.

**Fig 6:**
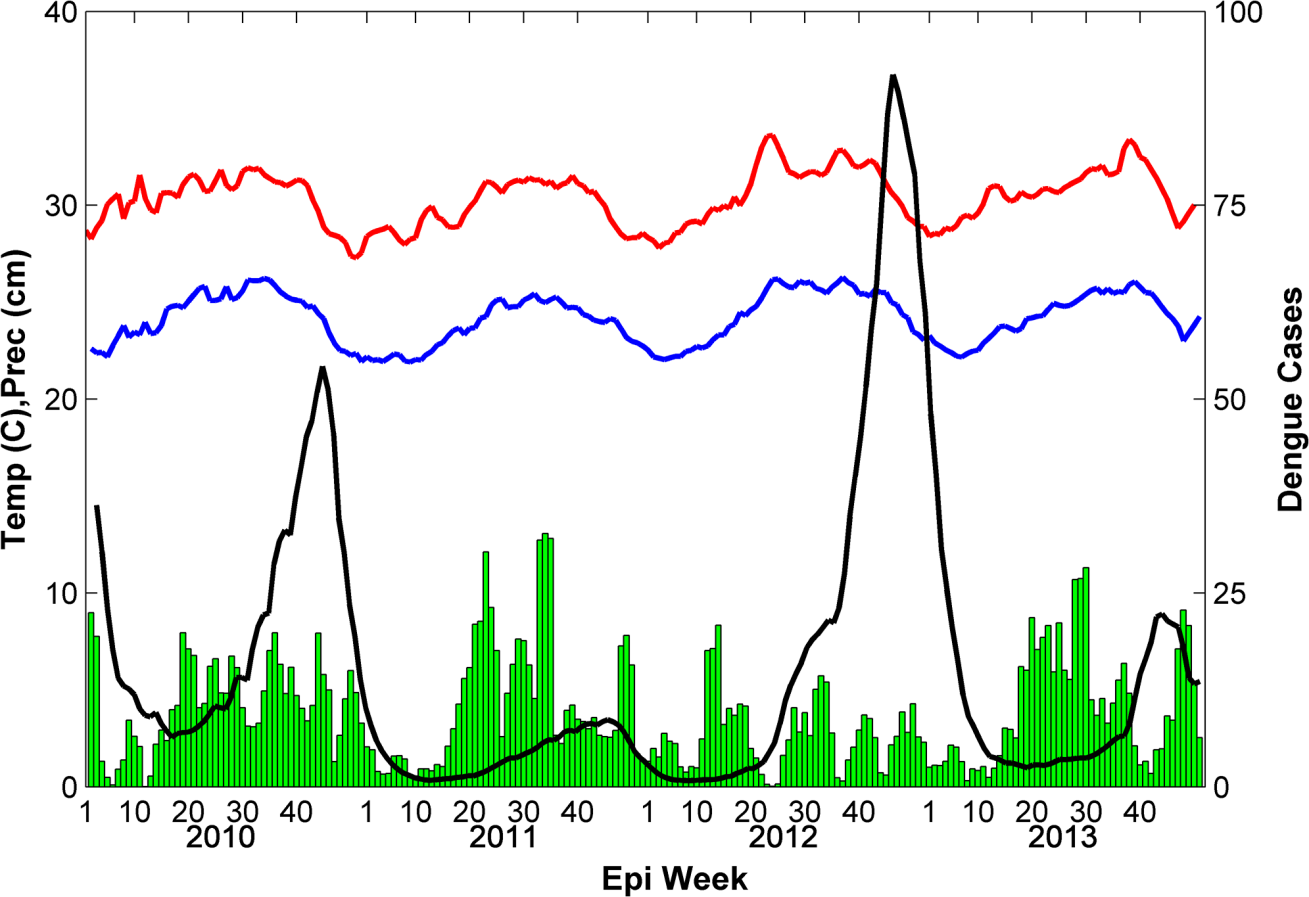
Weekly summarized climate and simulated DF cases over San Juan, 2010–2103. The green bars are weekly total precipitation, the red line is weekly mean maximum temperature, the blue line is weekly mean minimum temperature, and the black line is weekly total simulated DF cases.

Precipitation was highest in 2010 (227.5 cm) and 2011 (224.0 cm), with the onset of the rainy season starting a few weeks earlier in 2010 (Fig 6). Precipitation was by far the lowest in 2012 (140.3 cm), however, temperatures were warmest in 2012, especially during late fall (~1°C warmer in November). Precipitation was 216.3 cm in 2013 –only slightly drier than 2010 and 2011—but the timing of the precipitation was different, exhibiting a marked drop off during late summer/early fall.

## Discussion

Our results emphasize the complexity of the influence of weather on DENV transmission. While the best-fit “all years” simulations reasonably replicate the inter-annual variability of dengue cases from 2010–2013, resolving the intra-annual variability using the same parameter values over the whole time period is difficult. As the results for individual years suggest, it is likely that parameter values change over time, as do the primary influences that drive dengue transmission. For example, preferred habitat may change seasonally or with climate conditions [58]. Averaging over 96 simulations dampens some of the variability within individual years. The best-fit “all years” simulations also captured the inter-annual variability in the length of the dengue season, though the timing and peaks of transmission were sometimes asynchronous. The ability of DyMSiM to resolve the variability of the annual case load and season length may be useful for a variety of applications, such as studies focusing on potential climate change impacts on dengue incidence and seasonality (e.g., addressing the question of whether dengue incidence may become higher or lower, or whether the season will be extended under climate change scenarios).

The best-fit models for individual years accurately resolved the intra-annual variability for 2010, 2011, and 2012 (r^2^ = 0.90, 0.83 and 0.94 respectively). Parameters often changed in response to meteorological variability. Optimizing the DyMSiM parameters for single years may be useful for examining the causality of seasonal trends in DF case numbers. This type of simulation could also be used for short term predictions by selecting parameter values based on currently available case data and then running simulations for forthcoming weeks using weather forecast data. Alternatively, building a databank of epidemic profiles based on model results could provide a collection of possible scenarios that could occur given present conditions.

The timing of the onset of the dengue season and peak for year 2013 was not well simulated even with parameters optimized specifically for the year, illustrating the sensitivity and complexity of the disease system. The model predicted a rise and peak in dengue cases more than 50% higher and five months later. Many or all of the components of the virus’ ecology are constantly changing and their responses to external factors (such as weather) are situation dependent. Meteorological conditions may not have had a strong influence on intra-annual variability in 2013. A number of other factors not included in the model may have dominated transmission that year such as changing patterns of herd immunity to the specific circulating dengue serotype(s), introduction of a new variant of a serotype earlier in the season, implementation of intervention methods such as novel source reduction of habitats, or other human related factors such as extensive use or reduction of water storage. While it is beyond the scope of this paper to determine which of these factors may have been influencing the transmission, a variant of one of the four serotypes could have been introduced early in the season but all four serotypes had been circulating previously in Puerto Rico [59]. Shifting herd immunity could play a role in reducing the overall level of reported cases but should not greatly influence intra-annual variability of reported cases. The greatest increase in immunity likely occurred during 2010 and 2012 when Puerto Rico experienced high levels of transmission (Fig 2). Additionally, if higher levels of herd immunity played a role, it would be expected that there would be a delay in the onset of cases while we observed that reported case data peaked much earlier than the modeled cases. It is also possible given the high level of transmission in 2012, that this early peak in 2013 was propagated transmission from the previous outbreak; with initial transmission into the general population which spread to a smaller adjacent geography. Propagated transmission of dengue has been observed in other areas of the world [60]. Further exploration into the potential factors influencing the patterns observed in 2013 are warranted. Changes in intervention strategies or patterns of container habitats could change DF dynamics by reducing or enabling transmission despite climatic conditions. For example, the model over-predicts DF cases late in the year. This could be due to fewer container sources, reductions in susceptible hosts, or increased use of pesticides.

In the present study we were able to rank the best simulations based on their fit to previously collected case data considered to be reliable. However, accurate case data may not be available in many locations, preventing the selection of parameters based on best-fit simulation. Therefore, we also tested the fit of the ensemble median of all 9600 simulations to the raw case data, in order to determine whether reasonable accuracy could be attained by assuming a wide range of parameters without any a priori knowledge about a given location. The r^2^ values for 2010, 2011, 2012, and 2013 were 0.77, 0.64, 0.05, and 0.12 respectively. The results indicate that the model is robust during certain years (2010 and 2011), but specific parameter values may be essential for anomalous conditions such as the extremely dry period during 2012 and early 2013. Interestingly, parameter values that produced the top 1% of simulations are not always consistent with those that produced the best simulations on average across all 9600 runs. For instance, on average the worst simulations occur with high values of container habitat area, however, many of the best simulations for 2010 and 2011 use a high value. A similar phenomenon occurs for the adult daily survival rate. Further analysis in future studies may reveal parameterizations that are robust over a wider array of conditions and locations. Currently, caution should be taken when interpreting model results that do not include either parameter selection by comparison with reported case data or data about location specific parameters such as container magnitude and type. It is recommended that parameter values producing the best simulations on average be used if there is no a priori training data.

Variations in some DyMSiM parameter values had a particularly important influence on model performance. Parameter response to meteorological variability is best exemplified in 2012, during which simulations with a higher amount of human managed water sources were best able to resolve dengue transmission. This is almost certainly a consequence of the drier conditions which made rain-dependent water sources ineffective as immature habitats. A study conducted by Barrera et al. [61] monitored adult and immature mosquito populations and habitats in San Juan and discovered that vector populations were sustained even in drier conditions through human managed water sources. Although precipitation had an obvious influence on population dynamics, human managed water sources also proved to be important habitats for the *Ae*. *aegypti* vector. We hypothesize that as has been seen in other areas [21,22] human managed water sources likely became more important habitats during the drier conditions of 2012 either because there was less precipitation to fill open containers or because of a corresponding increase in man-filled containers for water storage in response to drier conditions. Additionally, particular values were shown to be of less importance for the top 1% of simulations, including the host infection probability and container height.

**Fig 7:**
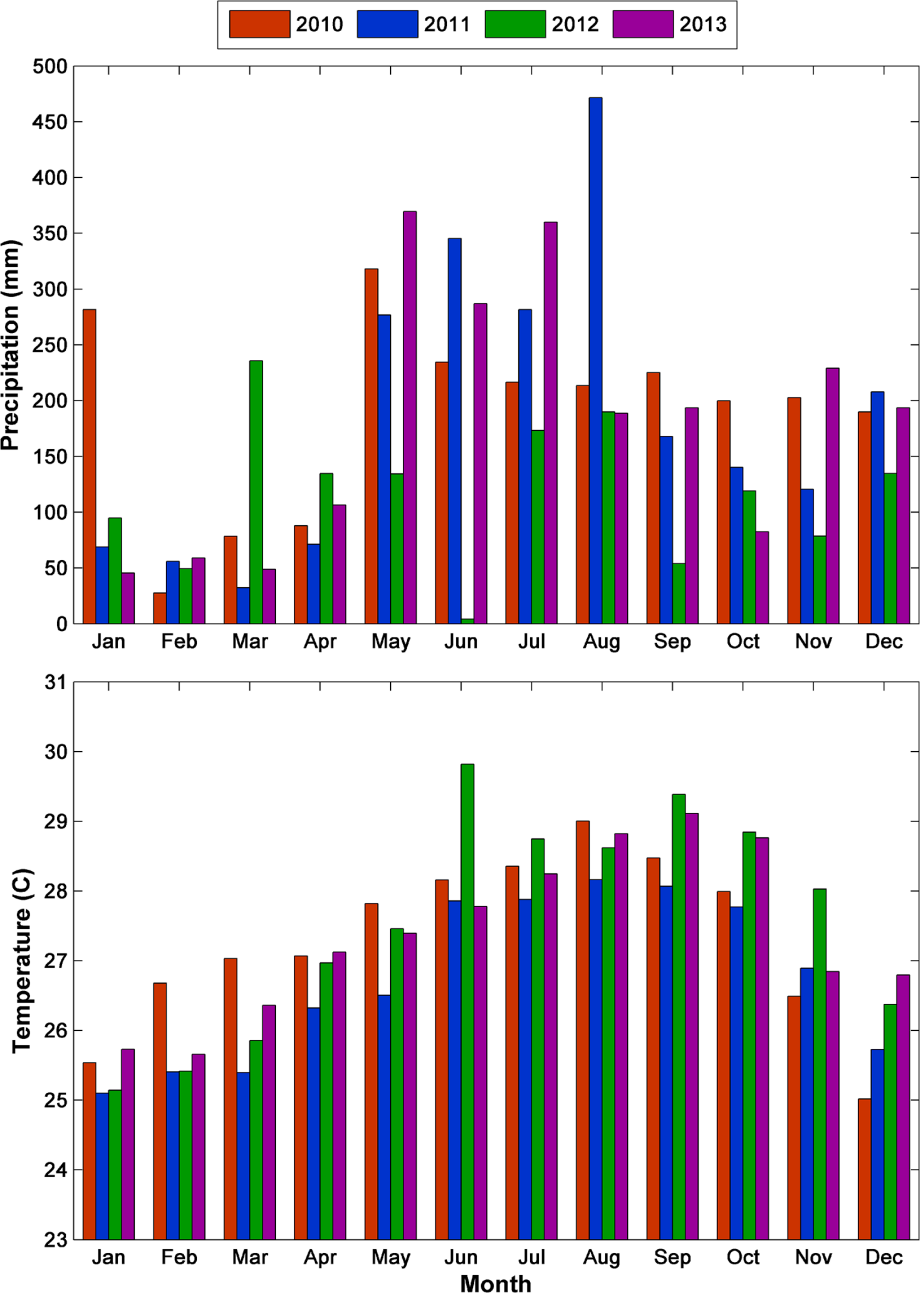
Monthly total precipitation (top) and mean temperature (bottom) by year. The highest incidence of DF cases occurred in 2010 and 2012 during which temperatures were highest in spring during 2010 and fall and early winter during 2012. The year 2011 had both the lowest DF incidence and the lowest temperatures. The year 2013 had moderate precipitation and temperatures but low DF incidence.

While precipitation patterns likely contribute to oviposition habitat selection by *Ae*. *aegypti* adults and consequently affect intra-annual transmission dynamics, variations in temperature may be largely responsible for fluctuations in annual DF case-loads, especially given the sensitivity of the length of the EIP to temperature. While average monthly temperatures generally vary between 25°C and 30°C, this translates to an almost four day difference in the length of the EIP (14.6–10.8 days) which can accelerate transmission. For example, the comparatively warm temperatures during El Nino in the winter and spring of 2010 (Fig 6) could have facilitated early and more rapid transmission of the virus (shown in the reported and simulated case data) by accelerating the mosquito life cycle and shortening the EIP. Conversely, the cooler temperatures persisting during La Nina throughout 2011 may have limited transmission, despite the wet conditions, resulting in fewer DF cases (Fig 7). In 2012, temperatures were relatively cool during the first five months of the year but were among the warmest across all years for the final seven months, especially during late fall (Fig 7). This explains the late seasonal peak in reported and simulated DF cases and the overall high annual incidence. In another Puerto Rico study, Jury [62] also observed that yearly fluctuations in DF caseloads were related to temperature while the timing was largely regulated by precipitation patterns. These results illustrate how nonlinear relationships between temperature, the mosquito lifecycle, and the EIP can generate considerable shifts in DENV transmission dynamics from small temperature differences. Interestingly, despite moderate temperatures and precipitation, 2013 experienced low levels of DENV transmission in San Juan (Fig 7). Shifting spatial patterns of herd immunity may explain the relatively low transmission in San Juan during 2013, especially since other parts of Puerto Rico experienced high case numbers and meteorological factors did not appear to strongly influence transmission.

Although there are similarities to other models, DyMSiM differs in important ways. CIMSiM, for example, produces mosquito population estimates over a 1 ha area which interact with human populations in DENSiM to simulate virus transmission between the two populations [34]. These models, though very comprehensive, require a great deal of site-specific information and training with many preset parameters. Parameter values in DyMSiM, however, can be evaluated and selected using ensemble simulations and locally reported case data with relative ease. DyMSiM has also been parameterized using newly acquired and synthesized data on vector development and virus EIP (S1 Table). These and other models may complement each other; by comparing strengths and weaknesses for specific applications and research goals. And, when used in conjunction, simulation results can be synthesized to determine the variance and confidence levels in model predictions and increase the accuracy of results [38].

### Limitations

While we used the best available information from the literature to specify values of the DyMSiM parameters, some values are based on the results of only a few studies or were reasonable estimates when quantitative results were unavailable. Papers that consolidated and considered data from multiple studies proved especially useful because they accounted for variations arising from conducting research on different colonies of mosquitoes and strains of virus or using different methods to answer the same question [17,39]. Often averages were used to enhance simplicity and interpretability of the model; yet, many parameters may have values across an unknown distribution surrounding their mean due to genetic and environmental variability. For example, genetic variations have been found to alter temperature regulation of West Nile virus transmission [63]. If a similar variation in temperature sensitivity exists between or within dengue serotypes it could be an important component to the severity of DF epidemics. We attempted to mitigate our limited knowledge of some parameter values by using a Monte Carlo approach when they were particularly difficult to ascertain. The model showed that some parameters, such as container habitat area and composition of containers, were particularly important for simulating transmission. The carrying capacity density was also important as it influenced the mosquito productivity within containers, and was directly related to their area. For example, simulations were degraded when using larger container areas, but this effect was lessened when using the lower maximum larval density value. The value of the background infection rate was also important when selecting the best fit models. This may indicate the importance of varying virus introduction or virulence between years. Vertical transmission of the virus was not considered in this model though it has been observed in the laboratory and field setting and could help maintain the virus during unfavorable conditions [64,65]. However, Adams et al. [66] found it did not have considerable importance in their model and the frequency of its occurrence in nature is still uncertain [67]. As more evidence is generated establishing links among meteorological factors, vectors, virus, and humans, parameter values can be better defined.

Some of the inconsistencies between modeled and reported case data may also be attributed to the nature of surveillance data. Because the surveillance system is passive, the reported case data are only an approximation of total DF incidence for the municipality. Incomplete or inaccurate reporting can arise due to sub-clinical infections, misdiagnosis, failure to report cases, differences between where virus transmission is reported and where it was acquired, and variability in the time between transmission of the virus, development of symptoms, and clinical visits. Relative increases in dengue reporting may be seen if there is increased media attention, circulation of a more severe strain, or increases in laboratory testing ability, etc. The serotype of the virus is often not reported but could be of particular importance if a high proportion of the population is immune to the circulating serotype. It is likely that not all cases are being reported to the CDC and, therefore, the reported case data is only a subset of the actual number of cases occurring in San Juan though it is difficult to estimate the amount of underreporting. The influence of under-reporting should be minimal if the patterns of reported cases represent the yearly variability even if the overall magnitude of reported cases is inaccurate. The largest bias in the calculation of the correlation between modeled output and reported case numbers would occur if there were significant changes to the reporting system or if there are interannual differences in reporting. There were no significant changes to the surveillance system protocol during this time period.

It is difficult to determine inter-annual variability in reporting, but there is the possibility for bias. For example, if individuals at the beginning of the season were more likely to seek care and be reported through the surveillance system, or if physicians were less likely to diagnose and report cases during typically lower periods of dengue, the shape of the curve might shift. Given the uncertainty of these types of scenarios, trying to determine the relative impact they may have on the correlation between model output and reported case numbers would be highly speculative. We acknowledge, however, that under-reporting of dengue is an important issue and therefore, the model output should not be interpreted as an estimate of actual dengue case numbers but as a representation of inter-annual variability of DENV transmission. If more reliable estimates of reporting bias and information on the temporal variability in asymptomatic cases or the reporting of symptomatic cases become available, it would be useful to compare numbers of modeled and reported dengue case data. Despite these limitations, the reported case data are currently the best metric available for tracking DENV transmission and have been used in other studies to represent levels of DENV transmission in Puerto Rico [4,61,68].

### Application of Results

The results of this study provide important information for dengue control. First, epidemics can occur during both wet and dry years. Dengue cases were comparatively high in 2010, one of the wettest years on record for San Juan, and in 2012, a drier-than-normal year in which the rains came later than average. The warmer winter and spring temperatures are likely responsible for the greater number of DF cases during 2010 while warmer fall and winter conditions in 2012 likely helped propagate dengue transmission later in the year. It is common for dry years to be hotter due to a combination of factors that include: 1) fewer clouds facilitating higher daytime temperatures and 2) less evapotranspiration freeing a greater amount of solar radiation for heating. Second, the timing of the dengue cases varies substantially from year-to-year and appears to be linked with the timing and magnitude of rainfall. In the wetter 2010 year, DF case numbers rose sharply after the onset of the rains (Week 10) and peaked near Week 30, whereas in the drier 2012 year the cases began later (Week 20) after a highly sporadic beginning to the rainy season, and gradually climbed to a much later peak near Week 48. In both instances, the onset of DF cases coincides with or begins shortly after the onset of the rainy season, and rises steadily due to the time required for the mosquito population to development in newly formed habitats and completion of the EIP. Lastly, despite a tropical climate, small fluctuations in temperature can have considerable effects on DENV transmission. Often tropical environments are thought to be warm enough for year-round transmission with precipitation being the limiting factor. Our results indicate that nonlinear relationships result in greater temperature impacts on DENV transmission than would be expected. Monitoring climate as a proxy of dengue risk may provide public health workers with a simple tool to prepare and execute transmission intervention methods and arrange testing and treatment protocols before the DF season peaks. Early warning systems can be developed based on the identification of rainfall and temperature patterns that promote rapid dengue transmission [69–72].

Dominant *Ae*. *aegypti* container habitat appeared to vary with precipitation patterns. During the wetter years, 2010 and 2011, simulations using a smaller container area and more rain dependent containers were ranked better; however, during 2012 simulations using a greater amount of human managed water sources ranked better. This suggests that *Ae*. *aegypti* inhabit rain filled containers when available, in addition to human managed water sources, but drier conditions may limit immature mosquito habitats to permanent (septic tanks, large water tanks, etc.) or human managed (animal drinking pans, plant trivets) water sources. This pattern has been observed in field studies [58]. From a public health perspective, the magnitude of precipitation in a given year may inform how to refine the message conveyed to the public about how to limit mosquito habitat. During wet years, mosquito control strategies should focus on eliminating old tires, buckets, and other items that can be filled via precipitation. Drier years should shift the focus to treating permanent or human managed water sources and covering stored water to prevent mosquito infestation. Further investigation of how weather and rainfall patterns influence human behaviors such as water storage are needed to determine if this could be incorporated into the model.

Understanding weather influences on DENV transmission is important in the context of climate change especially as DENV range expands in the Americas. Outbreaks in Hawaii, along the Texas/Mexico border, and in southern Florida indicate a reemergence of dengue in the U.S. [73–75]. Additionally, sero-prevalence studies indicate that dengue cases are likely underreported in the U.S. [73,75] due to subclinical infections, insufficient resources for testing, lack of sensitization of the medical community and patients seeking medical attention across the border in Mexico [75]. Although infrastructure differences, such as the prevalence of air conditioning, were shown to reduce transmission on the US side of the Mexico border in Laredo, autochthonous cases still occur [18]. Viral introduction in response to frequent travel across the border by residents may be a large source of infections [76] and weather may still have an important influence on transmission risk [20]. Whether DENV is endemic to an area or currently only poses a threat, understanding the influence of weather and climate on DENV ecology can facilitate strategies to prevent or mitigate transmission.

### Conclusions and Future Directions

Using known relationships between climate variables, *Ae*. *aegypti* dynamics, DENV replication and transmission, and a basic SEIR model within a modeling framework known as DyMSiM we were able to simulate inter-annual variability in DF cases in San Juan County, PR for the years 2010–2013. When optimizing the DyMSiM parameters for a single year, we were able to simulate intra-annual variability well for three of the four years. Meteorological factors were important influences on dengue transmission for 2010, 2011, and 2012 but not as important in 2013. Parameter values changed in response to meteorological conditions, illustrating the complexity of DENV ecology.

The results of this study quantify meteorological impacts on DENV transmission and demonstrate the variance in parameter values that result from changing environmental conditions. Rain patterns were especially important for determining the timing of epidemics and the primary habitat for immature *Ae*. *aegypti* vectors. Abnormally high temperatures have the potential to extend the transmission season (even during dry years) as exemplified in 2012. In general, temperature had the greatest influence on annual case numbers. The sensitivity of DENV ecology to meteorological variables and their interactions underscores the utility of process-based modeling when studying the impacts of climate variability and climate change on vector-borne disease. Information from this study can be used by public health officials to improve DENV transmission intervention strategies in San Juan, PR by way of advanced preparedness through model predictions and creation of more targeted vector control campaigns. As our understanding of the ecology of the virus and its thresholds improves, so will our ability to implement effective mitigation strategies against DENV transmission and reduce the disease burden on human populations.

Future research endeavors will seek to enhance model performance and utility. Validation of DyMSiM in other areas with longer term datasets would be useful in determining its ability to simulate DENV transmission in other environments and its ability to simulate DF incidence over long time periods. The deficit of long, reliable dengue surveillance records has forced training of dengue models over periods of less than 10 years and validation on one or only a few years [70,77,78]. Inclusion of serotypes would also enhance the model given that immunity to specific virus serotypes can affect transmission dynamics by limiting the susceptible population. The current SEIR model is simple but can still be used to examine the dynamics of dengue over short time periods. Incorporating surveillance of multiple virus serotypes, including short term cross-immunity, will be a priority in future research given the importance of immunological status on virus transmission dynamics. Additionally, the inclusion of social factors that influence population risk, such as migration, age structure, personal and household level prevention behaviors and other factors influencing vector-human contact, may provide further insights into the nature of DF epidemics. Finally, future work will concentrate on the possible impacts of climate change on dengue transmission. For instance, under climate change, winter temperatures may become suitable for completion of the EIP allowing DENV transmission to continue. Understanding the effects of future weather and climate conditions on DENV transmission is vital for limiting increases in incidence of disease and range expansion.

## Supporting Information

S1 Table Parameter values of simulations for San Juan County, Puerto Rico. Parameters (and their values) in bold were varied to perform the Monte Carlo simulations.
(PDF)

S2 Table Governing equations of mosquito and human populations. (PDF)

S1 Dataset Daily meteorological data from 2009–2013 for San Juan, PR. (CSV)

S2 Dataset Top 1% of simulations evaluated against reported dengue fever cases for 2010–2013. (CSV)

S3 Dataset Top 1% of simulations evaluated against reported dengue fever cases for 2010. (CSV)

S4 Dataset Top 1% of simulations evaluated against reported dengue fever cases for 2011. (CSV)

S5 Dataset Top 1% of simulations evaluated against reported dengue fever cases for 2012. (CSV)

S6 Dataset Top 1% of simulations evaluated against reported dengue fever cases for 2013. (CSV)
