# Supplementary material for: Meteorologically Driven Simulations of Dengue Epidemics in San Juan, PR
Source: PLoS Negl Trop Dis. 2015 Aug 14;9(8):e0004002. doi: 10.1371/journal.pntd.0004002 (PMC4537107; doi:10.1371/journal.pntd.0004002)
Supplement: S1 Table — Parameters (and their values) in bold were varied to perform the Monte Carlo simulations. (PDF) [file pntd.0004002.s001.pdf]

Parameter	Temp Range (°C)	Equation/Constant	Source(s)
Egg Development: E_d	T<14.5, T>36	0	Eisen et al. 2014
(proportion of stage completed per day)	14.5≤T≤31	0.0012*T - 0.0171	
	31<T≤35	-0.0002*T + 0.0268	
	35<T≤36	-0.0198*T + 0.7128	
Egg Survival: E_s	T≤0, T≥38	0	Eisen et al. 2014
(Proportion surviving each day)	0<T<15	0.071*T - .071	
	15≤T<32	0.98	
	32≤T<38	-0.0141*T^2 + 0.8278*T - 11.096	
Minimum Egg Hatching Temperature: E_m		14°C	Christophers 1960
Larva Development: L_d	T≤14, T>38	0	Eisen et al. 2014
	14<T<36	0.0076*ln(T)-0.0192	
(proportion of stage completed per day)	36≤T≤38	-0.0027*T+0.1041	
Larva Density Independent Survival: L_s	T≤4, T≥38	0	Eisen et al. 2014
(Proportion surviving each day)	4<T<15	0.0876*T - .3502	
	15≤T<35	0.98	
	35≤T<38	-0.1863*T+7.4581	
Larva Density Dependent Survival: L_{ds}	< Carry Cap	1	
(Proportion surviving each day)	> Carry Cap	(CC*Habitable Water)/(L(t) + P(t))	
**Carrying Capacity : CC**			Barbosa et al. 1972, Moore and
**(Maximum larvae/pupae per ml water)**		**0.5, 1.0**	Whitacre 1972
Pupae Development: P_d	T≤12, T≥39	0	Eisen et al. 2014
(proportion of stage completed per day)	12<T<33	.0014*T-.0155	
	33≤T<39	-0.0004*T^2+0.0286*T-0.4777	
Pupae Survival: P_s	T≤4, T>39	0	Eisen et al. 2014
(Proportion surviving each day)	4<T<15	-0.0068*T^2 + 0.2201*T -0.7794	
	15≤T<33	0.99	
	33≤T≤39	-0.1585*T+6.2021	
Ovarian Development: O_d	T≤20	0	Christophers 1960, Focks et al.
(proportion of stage completed per day)	T>20	Sharpe and DeMichele	1993
**Adult Daily Survival Rate: A_s**	T<1, T>40	.01	Muir and Kay 1998, Conway et al.
**(Daily survival Rate)**	1≤T≤40	**0.86, 0.88**	1974

Minimum Thermal Oviposition Limit: O_l		18°C	Christophers 1960
Adult Biting Rate: A_b<br>(Proportion of adults that feed)	T<17, T>39<br>17≤T≤39	0<br>-0.0066*T^2 + 0.3694*T - 4.2222	Christophers 1960, Author Observations
Fertilizaton Rate(Age: 1, 2, 3 days): A_f	T<17<br>T≥17	0<br>0.33, 0.5, 1.0	Christophers 1960
Egg Batch Size: E_b		100	Christophers 1960
EIP Progress: EIP<br>(Proportion of EIP complete per day)	T<21<br>T≥21	0<br>(1/(100.33*EXP(T*-0.077)))/24	Tjaden et al. 2013
Vector Infection Probability: I_v<br>(Probability of infection)	T<17<br>T≥17	0<br>-0.0023*T^2+0.1655*T-2.0568	Watts et al. 1987
Length of Exposed Stage (days): H_e		5	Kuno 1995, Chan and Johansson 2012, Nishiura and Halstead 2007
**Length of Infectious Period (days): H_i**		**5, 7**	Same at H_e
**Background Infection Rate: I_b**<br>**(Proportion of infectious humans)**		**0.00004, 0.00006, 0.00008**	**Sensitivity Analysis, CDC data**
**Host Infection Probability: I_h**	**T<21**<br>**21≤T≤32**<br>**T>32**	**0**<br>**(0.0022*T^2 - 0.0627*T + 0.6232)*0.5, 1**<br>**0.5, 1**	Tjaden et al. 2013
**Container Height: C_h**		**8cm, 12 cm**	Sensititivy Analysis
**Container Habitat Area (cm^2): C_a**<br><br>**(All 20 values were used sepeartely but results were grouped into five categories for analysis)**		**Low = 8E+6, 10E+6, 12E+6, 14E+6**<br>**Med Low = 16E+6 , 18E+6, 20E+6, 22E+6**<br>**Med = 24E+6 , 26E+6, 28E+6, 30E+6**<br>**Med High = 40E+6, 50E+6, 60E+6, 70E+6**<br>**High = 80E+6, 90E+6, 100E+6, 100E+6**	Sensititivy Analysis
**Container Habitat Composition: C_c**<br>**(Proportion Open Containers)**		**0.1, 0.3, 0.5, 0.7, 0.9**	Sensititivy Analysis
Evaporation (cm/day): Ev		Hamon's Equation	Hamon 1961
Daylight Hours: DL		See Reference	Forsythe et al. 1995
Population: P		395,326	US Census Bureau
Latitude: L		18.43° N	Weather Station Location

**T =Temperature, Eq = Equation*
