# Supplementary material for: Meteorologically Driven Simulations of Dengue Epidemics in San Juan, PR
Source: PLoS Negl Trop Dis. 2015 Aug 14;9(8):e0004002. doi: 10.1371/journal.pntd.0004002 (PMC4537107; doi:10.1371/journal.pntd.0004002)
Supplement: S2 Table — (PDF) [file pntd.0004002.s002.pdf]

Populations	Equation
Eggs: E(t)	$E(t-1) + \Delta A_{g1}(t-1)*E_b + \Delta A_{g2}(t-1)*E_b - \Delta E(t-1) - E(t-1)*(1-E_s(t-1))$
Larvae: L(t)	$L(t-1) + \Delta E(t-1) - L(t-1)*(1-L_s(t-1))*(1-L_{ds}(t-1)) - \Delta L(t-1)$
Pupae: P(t)	$P(t-1)+ \Delta L(t-1) - \Delta P(t-1) - P(t-1)*(1-P_s(t-1))$
Naïve Adults: A_n	$A_n(t-1)+\Delta P(t-1) - A_n(t-1)*A_f(t-1)*A_b(t-1) - A_n(t-1)*(1-A_s(t-1))$
Gravid Adults: A_{g1}	$A_{g1}(t-1) + A_n(t-1)*A_f(t-1)*A_b(t-1) -\Delta A_{g1}(t-1) - A_{g1}(t-1)*(1-A_s(t-1))$
Questing Adults:A_q	$A_q(t-1) + \Delta A_{g1}(t-1) + \Delta A_{g2}(t-1) - A_q(t-1)*(1-A_s(t-1)) - A_q(t-1)*A_b(t-1)$
Gravid Adults: A_{g2}	$A_{g2}(t-1) + A_q(t-1)*A_b(t-1) - \Delta A_{g2}(t-1) - A_{g2}(t-1)*(1-A_s(t-1))$
Infected Adults: A_i	$A_i(t-1) + A_n(t-1)*A_f(t-1)*A_b(t-1)*I_v(t-1)*H_{ip}(t-1) + A_q(t-1)*A_b(t-1)*I_v(t-1)*H_{ip}(t-1) - A_i(t-1)*(1-A_s(t-1))$
Human Suseptible: H_s	$H_s(t-1) - I_h(t-1)*A_q(t-1)*A_{ip}(t-1)*A_b(t-1)*H_{sp}(t-1)$
Human Exposed: H_e	$H_e(t-1) + I_h(t-1)*A_q(t-1)*A_{ip}(t-1)*A_b(t-1)*H_{sp}(t-1) - \Delta H_e(t-1)$
Human Infectious: H_i	$H_i(t-1) + \Delta H_e(t-1) - \Delta H_i(t-1)$
Human Recoverd: H_r	$H_r(t-1) + \Delta H_i(t-1)$
**Population Proportions**	
Infectious Mosquitoes: $A_{ip}(t)$	$\Delta A_i(t-1) / (A_{g1}(t-1) + A_q(t-1) + A_{g2}(t-1))$
Infectious Humans: $H_{ip}(t)$	$H_i(t-1) / (H_s(t-1) + H_e(t-1) + H_i(t-1) + H_r(t-1))$
Susceptible Humans: $H_{sp}(t)$	$H_s(t-1) / (H_s(t-1) + H_e(t-1) + H_i(t-1) + H_r(t-1))$
**Water Volumes**	
Human Managed: W_h	$W_h(t-1) + Pr(t-1)*C_a*(1-C_c) - Ca*(1-Cc)*Ev(t-1)*(1/5)$
Open Containers: W_o	$W_o(t-1) + Pr(t-1)*C_a*C_c - C_a*C_c*Ev(t-1)$

Note: If $H_{ip} < I_b$ then I_b replaces H_{ip}

Δ denotes members that have completed the stage

ΔA_{g1} and ΔA_{g2} assumes water is available to lay eggs on/around

Pr = Precipitation

W_h and W_o will no longer fill once they meet the maximum capacity ($C_a*(1-C_c)*C_h$ and $C_a*C_c*C_h$)
